# Study on Spectrum Shifting and Pulse Splitting of Mode-Locked Fiber Lasers Based on NPR Technology

**DOI:** 10.3390/nano14090739

**Published:** 2024-04-23

**Authors:** Zhenhua Hao, Yu Hu, Siyu Zhou, Jinhui Liu, Xiaohui Li, Yishan Wang, Cunxiao Gao

**Affiliations:** 1 State Key Laboratory of Transient Optics and Photonics, Xi’an Institute of Optics and Precision Mechanics, Chinese Academy of Sciences, Xi’an 710119, China; haozhenhua21@mails.ucas.ac.cn (Z.H.); huyu@opt.ac.cn (Y.H.); zhousiyu22@mails.ucas.ac.cn (S.Z.); liujinhui21@mails.ucas.ac.cn (J.L.); yshwang@opt.ac.cn (Y.W.); 2 University of Chinese Academy of Sciences, Beijing 100049, China; 3 School of Physics and Information Technology, Shaanxi Normal University, Xi’an 710062, China; lixiaohui@snnu.edu.cn

**Keywords:** mode-locked laser, nonlinear polarization rotation, spectrum shift, multi-pulse

## Abstract

We conducted a systematic investigation into the spectral and pulse characteristics of C and L-band Nonlinear Polarization Rotation (NPR) mode-locked fiber lasers effectively employing nonlinear polarization rotation technology. In our experimental setup, we achieved a stable mode-locked state at 1560.076 nm, exhibiting a 3 dB spectral bandwidth of 9.1 nm. As the pump power increased, we observed spectral shifts accompanied by shifts in the first Kelly sideband and the generation of new Kelly sidebands. In this paper, the phenomenon of spectral deviation is elucidated through the interplay of self-phase modulation, group velocity drift, and polarization-dependent isolator (PD-ISO) filter effect, with an analysis of the formation and deviation of Kelly sidebands. Notably, spectral shift persisted even when the pump power exceeded 200 mW. However, continuous pump power escalation led to soliton splitting, resulting in the formation of new soliton beams. Based on the simultaneous generation of spectral shift and pulse splitting, our study contributes to an enhanced understanding of soliton dynamics in ultrafast fiber lasers and lays a foundation for the application of high-repetition-frequency harmonic mode-locked lasers with tunable wavelengths.

## 1. Introduction

Nonlinear Polarization Rotation mode-locked fiber lasers, as important laser devices, have a wide range of applications in optical communication, optical measurement, spectral analysis, and other fields. In fiber mode-locked lasers, the use of saturable absorbers (SESAM), NPR, and nonlinear amplifying ring mirrors (NALM) [1] are common mode-locked methods. In addition to the above methods, there is also a mode-locking method based on the properties of heavily doped active fibers without the use of additional saturable absorbers [2,3,4]. Among these methods, NPR mode locking, which replaces saturable absorbers with a combination structure of polarization controllers, nonlinear fibers, and isolators, offers advantages such as wide spectral coverage, narrow output pulses, and the capacity to withstand high pulse energy. However, due to the reliance on non-polarization maintaining single-mode fibers, NPR mode-locked lasers are typically deployed in relatively stable environments.

Over the past few decades, relying on the inherent filtering effect of NPR mode locking technology and the minimal need for modulation devices, studies have revealed various pulse mode-locking operations in C and L-band fiber lasers in experiments [5,6,7]. These operations encompass conventional mode-locked solitons, bound-state solitons, multi-pulse phenomena, and dissipative soliton resonance [8]. Some of these pulses exhibit distinct temporal profiles, including vector solitons, dark pulses [9], H-shaped pulses [10,11], step-shaped pulses [12,13], and chair-shaped pulses [14,15,16]. While numerous shaped pulses have been reported, it is anticipated that novel pulse profiles remain to be discovered and studied. Comparable transposable pulses and dissipative soliton resonances have been documented at 1.0 μm and 1.5 μm [17,18]. Studies have revealed that in fiber mode-locked lasers, single solitons and multiple solitons can be formed by adjusting the polarization controller and pump power, yet limited research has addressed spectral shift in the context of multiple soliton pulses [19]. Furthermore, harmonic mode-locking technology [20,21,22,23,24,25] utilizing pulse splitting is capable of generating high-frequency signals without the need for an RF source and modulator, thereby leading to significant cost reduction and simplification of the system structure. The multi-pulse generation process can be easily implemented in the study. In the absence of a band-pass filter in the cavity, when the pulse width is narrow enough to be limited by the gain bandwidth, it splits into two pulses. The frequency components of the multi-pulse propagating in the strong pumping gain medium also change due to the existence of nonlinear effects and PD-ISO in NPR fiber lasers. The simultaneous generation of spectral shift and pulse splitting is highly significant for the realization of tunable wavelength harmonic mode-locked lasers.

This paper introduces a mode-locked fiber laser based on NPR mode-locking technology. The laser’s mode-locked state is achieved through a combination of intracavity fiber, polarization controller (PC), and PD-ISO. By adjusting the pump power, stable mode locking and multi-soliton states within the cavity can be achieved. Various pulse mode-locking phenomena, including conventional mode-locked solitons and multi-pulse states, were observed and systematically analyzed.

## 2. Experimental Setup

**Figure 1:**
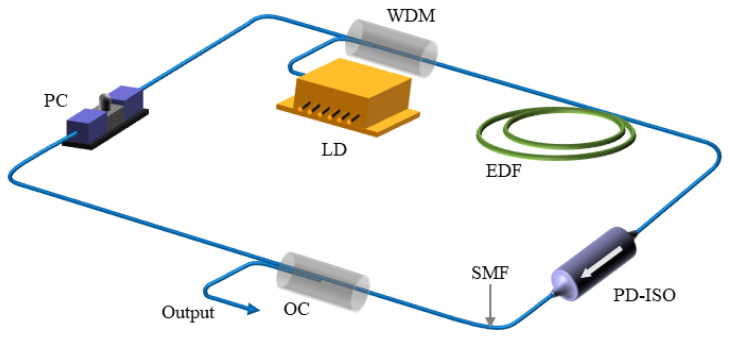
Schematic diagram of experimental setup. WDM: wavelength division multiplexer; OC: output coupler; EDF: erbium-doped fiber; PC: polarization controller; PD-ISO: polarization-related isolator.

Figure 1 shows the structural schematic of the mode-locked fiber laser within the experimental setup. A 980 nm laser serves as the pump source, with the pump light coupled into a 21 cm-long erbium-doped fiber (SM-ESF-7/125, Nufern, East Granby, CT, USA) via a 980/1550 wavelength division multiplexer (WDM). The amplified laser traverses PD-ISO, a 90/10 coupler, and PC before returning to the WDM. The PD-ISO (center wavelength 1560 nm, 40 nm working bandwidth) optimizes the polarization state of light, filters light, and ensures unidirectional light transmission. The 90/10 coupler separates 10% of the optical power from the cavity for measurement, while the remaining power cycles back through the cavity for subsequent rounds. The PC controls the polarization state of the laser within the cavity to achieve mode-locked operation. The PC, single-mode optical fiber, and PD-ISO collectively function as a saturable absorber.

NPR mode-locked fiber lasers utilize the nonlinear polarization rotation effect to achieve laser mode-locked output. As the laser propagates through the fiber, it experiences nonlinear effects such as self-phase modulation and cross-phase modulation. Variations in the polarization directions of light with varying intensities result in differing transmittance levels when passing through the PD-ISO, akin to the behavior of saturable absorbers. Adjusting the PC allows for the transmission of high-intensity light while selectively blocking low-intensity light, leading to the loss of the pulse’s edge due to decreased intensity and the smooth passage of the central region with higher intensity. This cyclical process enhances gain and ultimately results in pulse narrowing, culminating in the generation of ultra-short pulses and the achievement of mode locking. Additionally, a PC is added post-output to finely tune the polarization state of the output light and regulate its intensity. All fibers and optical components used are standard non-polarization-maintaining single-mode fibers (SMF-28e). The total cavity length measures 4.7 m, with a total group velocity dispersion of −0.21 ps^2^, indicating the formation of conventional solitons within the cavity.

The experiments employed spectral analyzers (OSA, AQ6370D, Yokogawa, Tokyo, Japan) for spectral measurements, digital storage oscilloscopes (SDS5104X, Siglent, Shenzhen, China) to monitor pulse sequences, and spectral analyzers (E4447A, Agilent, Santa Clara, CA, USA) for measuring and analyzing pulse repetition frequency signals.

## 3. Experimental Results and Discussion

**Figure 2:**
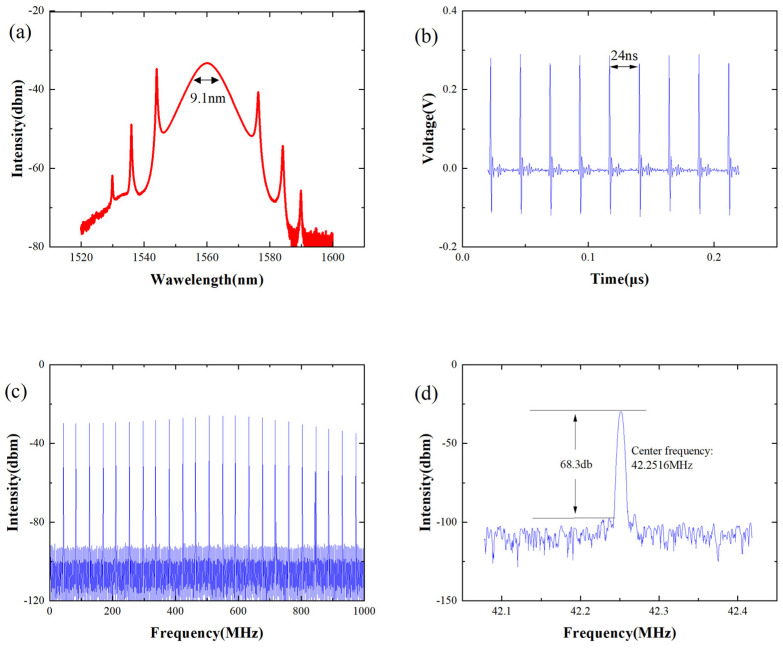
Pulse characteristics of stable soliton states. (**a**) Spectra; (**b**) pulse sequence; (**c**) spectrum sequence; (**d**) fundamental frequency signal.

At a pump power of 70 mW, adjusting the PC angle and screws appropriately results in the laser operating in a stable mode-locked state. By maintaining a fixed PC setting and increasing the pump power to 80 mW, the effects of intracavity dispersion-induced broadening and nonlinear effects-induced compression counterbalance each other [19,20], leading to a constant pulse width and shape, thus forming a soliton pulse that retains its shape and intensity during transmission. The laser self-starts, with the corresponding pulse characteristics depicted in Figure 2. The spectrum exhibits a conventional soliton profile centered at 1560.076 nm with a 3 dB spectral width of 9.1 nm (see Figure 2a). A stable pulse sequence with a pulse interval of 23.7 ns was obtained using an oscilloscope (see Figure 2b). Subsequently, the pulse repetition frequency signal was measured (see Figure 2c), revealing a repetition frequency of 42.2516 MHz and a fundamental frequency signal-to-noise ratio of 68.3 dB (see Figure 2d).

**Figure 3:**
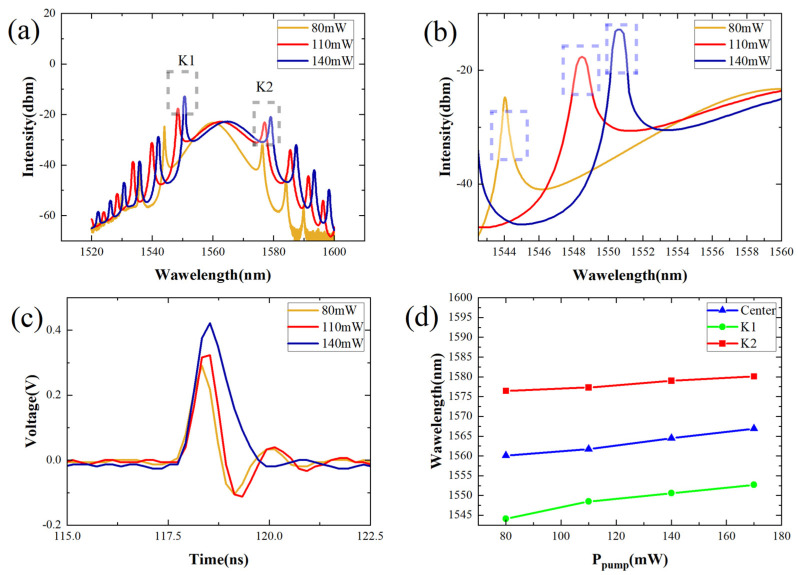
The spectrum and the pulse characteristics without changing the PC. (**a**) Increase the pump power to 80 mW, 110 W, and 140 mW for the output light spectrum; (**b**) enlarged first Kelly sideband K1; (**c**) single pulse sequence; (**d**) the relationship between pump power and the position of center wavelength and Kelly sidebands.

As the pump power continues to increase, the pulse characteristics change and spectrum shift occurs, as shown in Figure 3. In Figure 3a, if the pump power is increased to 130 mW and 180 mW, respectively, we can obtain the spectrum of the laser at this time. Obviously, new sub-sidebands will appear on the left side of the spectrum, and the number of Kelly sidebands increases. At the same time, the first-order Kelly sidebands on both sides are clearly red-shifted (see Figure 3b). On the oscilloscope, we can further observe that the shape of a single pulse has changed, with the pulse becoming wider and higher (see Figure 3c). In Figure 3d, we can see the relationship between the pump power and the position of the center wavelength and Kelly sidebands.

The spectral shift can be explained by the nonlinear effect and the filter effect of PD-ISO on the pulse. Typically, group velocity dispersion (GVD) causes the leading edge of the pulse to undergo a blue shift and the trailing edge to undergo a red shift due to its effect of making the leading edge very dense and the trailing edge very sparse. This is because the speed of the high frequency component is greater than that of the low frequency component. In contrast, self-phase modulation (SPM) will induce a positive chirp, resulting in a red-shifted pulse front and a blue-shifted rear edge, which reduces the frequency difference between them and leads to pulse compression. The red-shift component travels faster than the blue-shift component. Ultimately, SPM causes pulse narrowing, partially compensating for dispersion and allowing stable solitons to form within the cavity [26]. PD-ISO in the cavity can be likened to a bandpass filter, allowing the peak part of the pulse to pass through while attenuating the edge. However, changes in pump power cause a red shift in the frequency component of the pulse peak, effectively shifting the “bandpass filter” range towards longer wavelengths. The selection of pulses in the time domain influences wavelength variation in the spectrum.

The Kelley sideband arises from the presence of periodic amplification and loss perturbations in the cavity during soliton propagation in fiber lasers [27]. As solitons propagate, they emit dispersive waves to adapt to these external disturbances. If the phase difference between the dispersive wave and the soliton wave at certain wavelengths is an even multiple of π, it results in the formation of a Kelly sideband [28]. Higher pump powers amplify the energy of second-order and third-order dispersion waves, intensifying their impact on Kelly sideband shifts. Conventional solitons within the resonant cavity periodically interact with the PC, PD-ISO, and nonlinear fibers. These periodic disturbances prompt solitons to adapt their parameters, emitting dispersion waves. Interference may occur before dispersion waves dissipate completely, resulting in the generation of Kelly sidebands at positions where interference strengthens. As pump power reaches sufficiently high levels, the interplay of gain, loss, dispersion, nonlinear effects, and other factors leads to unstable dispersion waves, causing shifts in interference positions [29]. The interaction between asynchronous resonant dispersion waves and solitons induces changes in pulse intensity, triggering soliton oscillations and synchronous resonant dispersion wave alterations that shift the positions of Kelly sidebands. Following spectrum shift, high-frequency components exhibit faster transmission speeds and reduced delays, while low-frequency components experience slower transmission speeds and increased delays within the anomalous dispersion region of optical fibers. This separation of high and low-frequency components leads to pulse width expansion.

**Figure 4:**
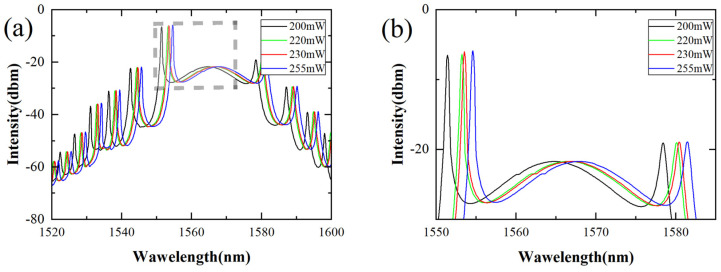
(**a**) The spectrum when increasing the pump power to 200 mW, 220 W, 230 mW, and 250 mW; (**b**) enlarged first Kelly sideband.

To verify the occurrence of spectrum shifts and explore pulse characteristics under different conditions, we incrementally increased the pump power from 200 mW to 255 mW to observe the spectrum (see Figure 4). Shifts in spectra and Kelly sidebands were observed. The spectrum at a pump power of 200 mW (see Figure 4a) displayed a central wavelength of 1564.5 nm and a 3 dB bandwidth of 8.8 nm. The first Kelly sideband was enlarged (see Figure 4b), and the shift in the Kelly sideband was clearly evident. Notably, we observed that as pump power increased or decreased, the number of pulses also correspondingly increased or decreased.

**Figure 5:**
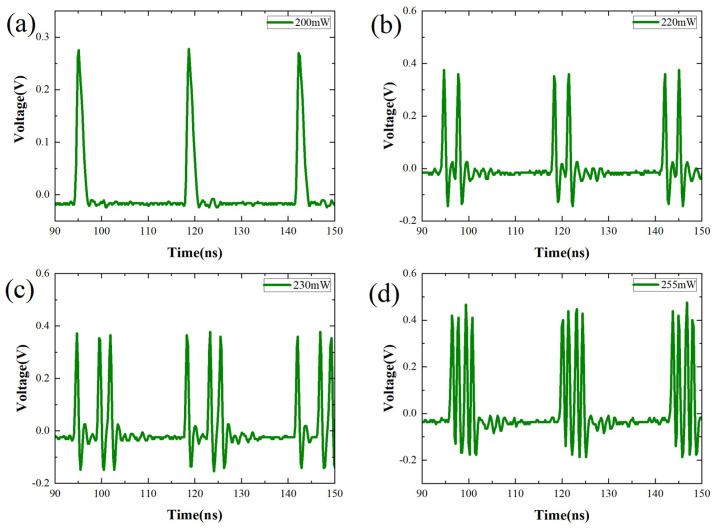
Pulse sequences with different numbers of solitons under pump powers ranging from 200 mW to 255 mW.

When the pump power exceeds 200 mW, a notable phenomenon of pulse splitting emerges. As illustrated in Figure 5, a single pulse undergoes division into multiple pulses. Through the experiment, diverse multi-soliton states were achieved by elevating the pump power beyond 200 mW. Specifically, as the pump power escalates from 200 mW to 255 mW, solitons are sequentially generated, manifesting as 1, 2, 3, and 4 pulses, as depicted in the figure. Beyond 255 mW, the pulse sequence transitions into an irregular state.

The comparison in Table 1 shows the spectral shift results as the pump power increases from 80 mW to 255 mW. It is evident that with the increase in pump power, the center wavelength of the spectrum shifts, corresponding to the shift in the Kelly sideband on both sides of the spectrum. The tunable range of the central wavelength is observed to be 1560.08 nm~1567.29 nm, with a maximum spectral shift distance of 7.19 nm.

It is worth noting that the chirp is close to zero due to the compensation of linear phase accumulation by anomalous dispersion fibers during the transmission of optical pulses. Multiple solitons gather together and propagate in the laser cavity. However, following soliton splitting, saturation absorption in the resonator causes split solitons to attract each other without remerging, eventually forming a soliton beam. Upon adjusting the pump power, the steadily formed soliton beam continues to split, ultimately producing four pulses. During this process, the peak power of the pulse increases and the nonlinear feedback effect in the laser cavity strengthens. However, traditional solitons typically have an energy of less than 0.1 nJ, leading to pulse splitting and rearranging when their peak value is too high due to the peak power limiting effect [30,31]. As a result of gain competition effects within the laser cavity, multi-solitons can reach a stable state with quantized final energy levels.

The peak-power-limiting effect in a passive mode-locked laser refers to the generation of multiple soliton pulses and the quantization of soliton energy due to the peak power limitation within the laser cavity. Research findings indicate that with a small linear phase delay bias setting, increasing pump power leads to higher peak power and a narrower pulse width of soliton pulses. However, this effect plateaus at a certain pump power, causing instability in the simulation window with weak background pulses. Slight increases in pump power result in the rapid formation of additional solitons through shaping weak background pulses, known as the “soliton shaping of dispersive waves”. Ultimately, all solitons reached a steady state with uniform pulse width and peak power. A further increase in pump power leads to the formation of multiple solitons, thus restricting the generation of light pulses with larger energies and narrower widths.

In the experiment, it was observed that as the pump power increases, the number of solitons also increases once the pulse peak reaches a certain threshold. New solitons are successively generated and multiple solitons are then coalesced to form a soliton bundle. However, when the pumping power exceeds a certain level, the laser transitions from its mode-locked state to randomly distributed chaotic multi-solitons. Therefore, by controlling the pump power within the range of 200 mW to 255 mW, it is possible to achieve a wavelength tunable multi-soliton pulse output ranging from 1564.17 nm to 1567.29 nm.

## 4. Conclusions

The article showcases an erbium-doped fiber mode-locked laser employing nonlinear fibers, polarization controllers, and polarization-related isolators as equivalent saturable absorbers to achieve mode-locking. The pulse evolution process can be observed by properly adjusting the cavity parameters, as well as the PC and pump energy. At 80 mW pump power, the laser achieves self-starting mode-locking, yielding stable conventional solitons with a pulse interval of 23.7 ns and spectral bandwidth of 9.1 nm. With the increase in pump power, spectral deviation occurs due to the influence of nonlinear effects on pulse frequency and PD-ISO filtering. In the experiment, as the pump power continues to increase to a certain threshold value while the PC state remains unchanged, pulse-splitting phenomena can be observed simultaneously with spectral deviation. The experiment demonstrates that altering the pump power not only induces spectral variations in NPR mode-locked lasers, but also leads to the formation of multi-soliton pulses. As the pump power increases from 200 mW to 255 mW, multiple solitons gradually emerge, and all solitons exhibit identical pulse characteristics in steady state. The presence of several solitons in the cavity is attributed to the quantization of soliton energy. Throughout this process, simultaneous shifts in the spectrum and the first Kelly sideband occur. The experiment demonstrates that, at specific pump power levels, it is feasible to achieve a tunable wavelength output of multi-soliton pulses. The findings in this study will contribute to further explorations of complex soliton dynamics in future C and L-band fiber lasers, while the observed spectral shift and pulse splitting phenomena will establish a solid foundation for the potential application of high-repetition-frequency harmonic mode-locked lasers with tunable wavelengths.

## Figures and Tables

**Table 1:** Comparison table of spectral shift results as the pump power increases from 80 mW to 255 mW.

Pump Power (mW)	Central Wavelength (nm)	Position K1 (nm)	Position K2 (nm)
80	1560.08	1544.21	1576.33
110	1561.91	1548.35	1577.82
140	1562.23	1550.44	1578.21
200	1564.17	1552.63	1578.93
220	1565.77	1553.87	1580.16
230	1566.53	1554.21	1580.83
255	1567.29	1555.14	1581.99

## Data Availability

Data are contained within the article.
